# Validity of Hip and Ankle Worn Actigraph Accelerometers for Measuring Steps as a Function of Gait Speed during Steady State Walking and Continuous Turning

**DOI:** 10.3390/s21093154

**Published:** 2021-05-01

**Authors:** Lucian Bezuidenhout, Charlotte Thurston, Maria Hagströmer, David Moulaee Conradsson

**Affiliations:** 1 Department of Neurobiology, Care Sciences and Society, Division of Physiotherapy, Karolinska Institutet, 141 83 Stockholm, Sweden; charlotte.thurston@ki.se (C.T.); maria.hagstromer@ki.se (M.H.); david.m.conradsson@ki.se (D.M.C.); 2 Academic Primary Health Care Centre, Region Stockholm, 104 31 Stockholm, Sweden; 3 Women’s Health and Allied Health Professionals Theme, Medical Unit Occupational Therapy and Physiotherapy, Karolinska University Hospital, 171 64 Stockholm, Sweden

**Keywords:** accelerometers, Bland–Altman plots, gait speed, interclass correlation coefficient, low frequency extension filter, Stepwatch

## Abstract

This study aimed to investigate the accuracy and reliability of hip and ankle worn Actigraph GT3X+ (AG) accelerometers to measure steps as a function of gait speed. Additionally, the effect of the low frequency extension filter (LFEF) on the step accuracy was determined. Thirty healthy individuals walked straight and walked with continuous turns in different gait speeds. Number of steps were recorded with a hip and ankle worn AG, and with a Stepwatch (SW) activity monitor positioned around the right ankle, which was used as a reference for step count. The percentage agreement, interclass correlation coefficients and Bland–Altmann plots were determined between the AG and the reference SW across gait speeds for the two walking conditions. The ankle worn AG with the default filter was the most sensitive for step detection at >0.6 m/s, whilst accurate step detection for gait speeds < 0.6 m/s were only observed when applying the LFEF. The hip worn AG with the default filter showed poor accuracy (12–78%) at gait speeds < 1.0 m/s whereas the accuracy increased to >87% for gait speeds < 1.0 m/s when applying the LFEF. Ankle worn AG was the most sensitive to measure steps at a vast range of gait speeds. Our results suggest that sensor placement and filter settings need to be taken into account to provide accurate estimates of step counts.

## 1. Introduction

Walking is the most common form and used marker of physical activity, where the number of steps per day is associated with health, e.g., cardiovascular health, dementia and future mortality risk [1,2,3]. Accelerometry is an established method for measuring steps [4], with the Actigraph GT3X+ (AG; ActiGraph Corp.) being one of the most commonly used accelerometers. AG is a small triaxial accelerometer (dimensions: 4.6 cm × 3.3 cm × 1.5 cm; weight: 19 g) that can be worn on different body positions (e.g., wrist, ankle and hip) and has a dynamic range of ±6G (1G = 9.81 m/s^2^). Additionally, AG has a long battery life and can continuously measure physical activity for up to six weeks at 30 Hz [5,6]. For the AG software to detect a step, the bandpass filtered vertical component (*y*-axis) accelerometer signal must exceed a proprietary amplitude threshold and cross the zero axis (i.e., positive and negative values) of the proprietary amplitude threshold [7].

Accelerometer device placement and gait speed are known to influence the step detection accuracy of accelerometers [8,9]. The hip is the most commonly used placement for measuring walking since the hip accurately reflects the center of mass of the body. However, since slow walking generally has low acceleration amplitudes and the amplitude decreases from the ground upwards, the signal measured at the hip might not be sufficient to cross the proprietary threshold for detecting a step. Previous studies have shown AG to accurately detect steps at a gait speed of >1.0 m/s [5,10], whereas few studies have investigated the sensitivity of AG to detects steps at different ranges of gait speeds < 1.0 m/s. Slow walking speeds have been linked to various movement disorders and high risk of morbidity and mortality [11]. Therefore, it is important to be able to accurately detect steps at low gait speeds (<1.0 m/s) to be able to identify individuals who have an increased risk of deteriorating health. This is especially important for people with disability (i.e., stroke or Parkinson’s) or the elderly, who often have compromised gait speeds. Hergenroeder et al. [1] showed the hip worn AG percentage agreement with observed step count to be <52% at gait speed ≤ 1.0 m/s and >85% at >1.0 m/s in older adults [1]. While it has been suggested that the accuracy of accelerometers to detect a step is inversely proportional to gait speed [5,8,12], it is unclear at which gait speed the sensitivity of hip and ankle worn AG starts decreasing. Furthermore, previous studies exploring the step detection accuracy of accelerometers have assessed straight walking, i.e., steady speed without changing the walking direction [1,13,14,15]. However, walking in everyday life is rarely performed during steady state; in fact, most walking bouts in daily life include four steps or less [16] and 50% of the steps executed each day incorporate turning steps [17]. Therefore, it is important that validation of accelerometer step detection accuracy also incorporates non-steady state walking (e.g., turning).

To take into consideration low frequency/amplitude movements, AG developed a low frequency extension filter (LFEF), which increases the sensitivity of the accelerometer signal at low intensity movements by decreasing the proprietary amplitude threshold. This allows for more accurate step detection during slow walking (<1.0 m/s) [6,14,18]. Although the LFEF has shown to improve step count accuracy during slow walking, studies by Wallen et al. [18], Toth et al. [19] and Feito et al. [13,14] showed a significant overestimation in daily steps taken in the free-living environment. Little is known at which range of gait speeds the LFEF should be applied to optimize the sensitivity of step detection. While previous studies [8,9,20] have shown the accuracy of AG to determine steps to be poor during slow walking, no previous studies have explored at what level the accuracy drops when walking straight and performing continuous turns.

This study aspired to determine the validity of the ankle and hip worn AG in healthy adults in a controlled environment before testing the validity of the accelerometers in people with a disability (e.g., those with neurological diseases) or elderly people. The aim of this study was to determine the validity of hip and ankle worn AG accelerometer for measuring steps compared to step counts measured with a reference ankle sensor in healthy adults. We especially investigated the accuracy of the AG accelerometer to detect steps across a large span of gait speeds during straight walking and walking with continuous turns. Additionally, we observed the step detection accuracy of the AG using the default filter (i.e., AG-DF) compared to the LFEF (i.e., AG-LFEF).

## 2. Materials and Methods

### 2.1. Study Participants

Thirty healthy participants (14 males, mean age ± standard deviation: 42 ± 13 years) with no ongoing or recent medical conditions affecting their gait participated in this cross-sectional study. The study was approved by the Regional Board of Ethics in Stockholm (2017/1626-31 and 2018/2524-32) and all participants gave written informed consent prior to participation.

### 2.2. Data Collection

Participants attended one gait assessment including two different walking tasks; walking straight and walking with continuous turns, since these conditions reflect different walking patterns occurring in everyday life. Prior to assessment, participants were fitted with two AG’s and one Stepwatch (SW) activity monitor. SW has been shown to be sensitive to detect steps over a range of gait speeds, especially during slow walking [21]. The position of the accelerometers was guided by Webber and St. John [8] with the AG accelerometers attached around the right hip (above the iliac crest) and left ankle (proximal to the lateral malleolus), and the SW reference sensor positioned on the right ankle (proximal to the lateral malleolus) [8]. The AG devices recorded time series acceleration data at a sampling rate of 30 Hz whereas the SW sensor records the number of steps per one second epochs. For the SW sensor, the participants height were entered into the Modus Health software and the following configuration was used: “no quick stepping”, “slow walking speed”, “rarely varying pace” and “gentle leg motion” [8]. The AG and SW devices utilize the local computer time to initialize the device timestamp. In order to synchronize the AG and SW devices, the local time on the local computer were reset to coordinated universal time approximately 15 min before the start of data collection for each participant. We are aware of the potential problems that might occur during data synchronization (i.e., clock drifting), therefore we validated the start and stop times for each trail by; (1) asking the participants to stand still between 10 and 20 s before and after each trail to delineate the devices step onset and offset; (2) manually recording the start and stop times from the local computer and (3) comparing the devices step onset and offset times to the manually recorded start and stop time for each trail.

**Figure 1:**
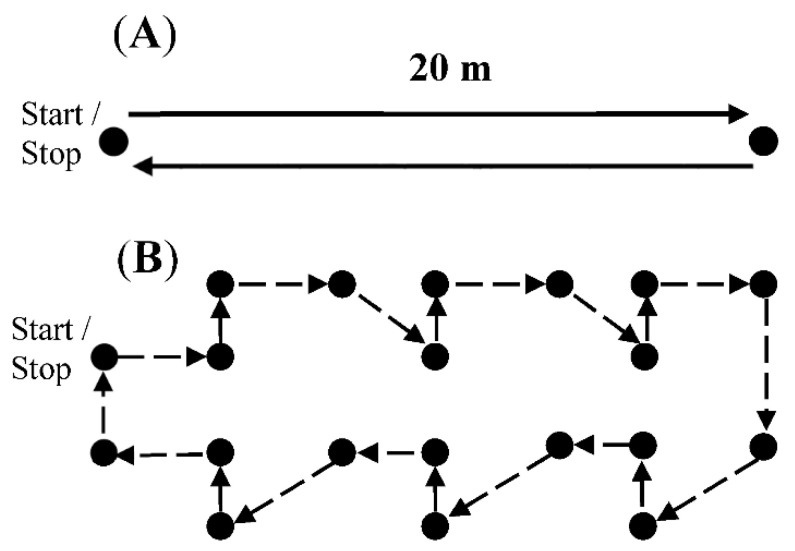
(**A**) Straight ahead walking. (**B**) Continuous turning. Each circle represents a cone and the direction of walking is shown by the arrows.

For straight walking, participants were asked to walk straight for a distance of 40 m with a 180 degree turn around a cone after 20 m (Figure 1A). For the walking with continuous turning trial, participants were instructed to walk through a maze (34 m distance) consisting of an equal distribution of 45 and 90 degree turns to the right and left (Figure 1B). Participants were instructed to start in their self-selective comfortable gait speed and gradually decrease their speed after each trail in order to achieve a good distribution of speeds for both walking conditions. Each participant performed between 10 and 15 trials per walking condition and we aimed to measure gait speeds between 0.2 and 1.6 m/s for straight walking and between 0.2 and 1.0 m/s for continuous turning. The time taken to complete each trail was measured with a stopwatch and immediately entered into a prearranged Microsoft Excel spreadsheet to calculate the average gait speed for each trail. The narrow gait speed range for turning reflects the nature of walking and continuous changing the direction, which often occurs at lower speeds. The start and stop time, manually counted steps and gait speeds were recorded for each trail by a trained investigator.

### 2.3. Data Analysis

The raw acceleration signal was bandpass filtered between 0.25 and 2.5 Hz to attenuate noise and artifacts and to extract physical movements [22]. Subsequently, the raw signal was digitized by a 12-bit analog to digital (A/D converter) at 30 Hz, which allows for 4096 levels of both positive and negative accelerations measurements (i.e., 2^12^ = 4096). Since the acceleration is both in the positive (acceleration towards the earth surface) and negative (acceleration away from the earth surface) direction, no movement (zero acceleration) is associated with the center of the A/D scale (i.e., A/D = 4096/2 = 2048). The positive and negative acceleration, which is proportional to the vector component of acceleration deviates the signal from the center value (zero acceleration). Subsequently, the AG software calculated the difference between the measured A/D reading and the center value and thereby only retaining the magnitude of the acceleration and removing the sign [23]. The magnitude of the AG signal is then converted to counts, where a count is defined as the acceleration amplitude crossing some AG proprietary amplitude threshold. The AG count data were then summed to one second epochs, i.e., sum of 30 counts per second, and exported to excel using the ActiLife 6 software (version 6.13.4). The number of steps was obtained using both the DF (0.25–2.5 Hz) [14] and the LFEF [14,24]. Subsequently, the AG step data were imported to Matlab where the start and stop times were used to delineate the total number of steps for each walking and turning trial. The SW data were exported to excel using the Modus Health (StepWatch4 RE 1.1.6) software. The SW output yields the timestamp at which each step was taken. The start and stop time were used to sum the number of steps for each trail of the two walking conditions. The total number of steps for each trail was grouped in four gait speed groups of 0.4 m/s for straight walking (i.e., 0.2–0.6 m/s, >0.6–1.0 m/s, >1.0–1.4 m/s and >1.4 m/s) and two gait speed groups for walking with continuous turning (0.2–0.6 m/s and >0.6–1.0 m/s). The gait speeds were stratified into 0.4 m/s in order to obtain a relatively good distribution of gait speeds samples within each group and to resemble gait speed categories of individuals who are dependent on others in activities in daily living and also have rehabilitation needs [25].

Statistical analyses were carried out using IBM SPSS v.27 software and the level of significance was set to 5%. In line with previous findings [8,9], the mean percentage agreement and two-way random inter class correlation coefficient (ICC_2,1_) between the manually counted steps and the SW activity monitor across all gait speeds was >99% and 0.99, respectively, for both straight walking and continuous turning. We therefore used SW as a reference for measuring steps in our study. The percentage agreement between the SW step count and the ankle and hip worn AG step count using the DF and the LFEF were calculated as: (AG step count/SW step count) × 100) for the gait speed groups [1,9]. The interrater reliability between the SW monitor and the AG devices was determined using the two-way random ICC_2,1_ for both filter settings. The strength of the ICC was classified as follows: <0.50 = poor; 0.50–0.75 = moderate; 0.75–0.9 = good and >0.90 = excellent [26,27]. Additionally, Bland–Altmann plots were used to describe the mean percentage bias between the SW and the AG devices [28,29]. We defined the mean percentage bias as: (the difference between the number of steps between AG and SW/mean number of steps between AG and SW) × 100 [29]. We also defined a mean percentage bias of <10% to be an acceptable agreement between the SW and AG devices.

## 3. Results

### 3.1. Number of Steps and Time Spent in Each Gait Speed Group for Straight Walking and Walking with Continuous Turning

For straight walking there was a lower mean number of steps and time spent in the >1.0 m/s gait speed groups (>1.0–1.4 m/s: 30 steps and 34 s and >1.4 m/s: 26 steps and 25 s) compared to <1.0 m/s gait speed groups (0.2–0.6 m/s: 54 steps and 103 s and >0.6–1.0 m/s: 36 steps and 51 s). For continuous turning the mean number of steps and time spent in the 0.2–0.6 m/s gait speed group was also higher (55 steps and 91 s) compared to gait speeds between >0.6 and 1.0 m/s (39 steps and 45 s). The lower time spent and the higher number of steps for walking with continuous turns compared to straight walking for the same gait speed ranges are indicative of the shorter walking distance (i.e., 34 m) and the nature of walking and turning, which often requires shorter steps.

### 3.2. Straight Walking

The mean percentage agreement for the ankle worn AG-DF was high (≥92%) at gait speeds > 0.6 m/s (Table 1) but dropped to 71% for gait speeds < 0.6 m/s. The ankle worn AG-DF showed moderate to good reliability (ICC_2,1_: 0.70–0.85) with the SW activity monitor at gait speeds > 0.6 m/s (Table 2) whereas the reliability was poor (ICC_2,1_ < 0.29) at gait speeds < 0.6 m/s. The ankle worn AG-LFEF showed a percentage agreement of ≥94% and moderate to excellent reliability (ICC_2,1_: 0.70–0.97) across all represented gait speeds. For the hip worn AG-DF, the percentage agreement was ≥92% at gait speeds > 1.0 m/s, with the percentage agreement decreasing rapidly at gait speeds < 1.0 m/s (Table 1). When the AG-LFEF was applied to the hip worn sensor, the percentage agreement was ≥96% for gait speeds > 0.6–1.4 m/s. While the hip worn AG-DF showed poor to moderate reliability (ICC_2,1_: 0.00–0.58) with the SW across all gait speeds, the agreement was moderate to good at all represented gait speeds when the LFEF was applied (Table 2).

**Figure 2:**
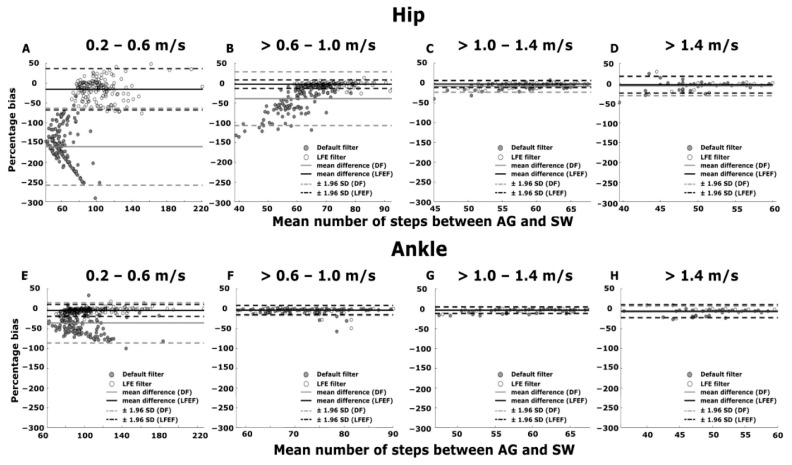
Bland–Altman plots for percentage bias between the hip worn AG and SW vs. the mean number of steps between the hip worn AG and SW using the DF and LFEF for gait speeds between (**A**) 0.2–0.6 m/s. (**B**) >0.6–1.0 m/s. (**C**) >1.0–1.4 m/s. (**D**) > 1.4 m/s during straight walking. Bland–Altman plots for percentage bias between the ankle worn AG and SW vs. the mean number of steps between the ankle worn AG and SW using the DF and LFEF for gait speeds between (**E**) 0.2–0.6 m/s. (**F**) >0.6–1.0 m/s. (**G**) >1.0–1.4 m/s. (**H**) > 1.4 m/s during straight walking. The grey and white filled circles represent the DF and LFEF, respectively. The grey and black solid line represents the mean percentage bias for the DF and the LFEF, respectively. The grey and black dashed line represents the ± 95% limits of agreement.

The Bland–Altman plots (Figure 2) showed a low mean percentage bias (−3.0–−8.0%) for both the AG-DF and AG-LFEF at gait speeds between >0.6 and >1.4 m/s (Figure 2F–H) and between 1.0 and >1.4 m/s (Figure 2C,D) for the ankle and hip worn AG, respectively. The mean percentage bias was high for the hip worn AG-DF for gait speeds ranging between 0.2 and 1.0 m/s (−40.0–−160.0%; Figure 2A,B) compared to the AG-LFEF. The high negative mean percentage bias is indicative of the number of steps being significantly underestimated by the AG sensors.

### 3.3. Walking with Continuous Turning

The percentage agreement for the ankle worn AG-DF was 95% for gait speeds between >0.6 and 1.0 m/s and 82% for gait speeds < 0.6 m/s (Table 1). The ankle worn AG-DF also showed good reliability (ICC_2,1_ = 0.89) at gait speeds between >0.6 and 1.0 m/s whereas the reliability was poor (ICC_2,1_ = 0.28) at gait speeds < 0.6 m/s (Table 2). When the AG-LFEF was applied to the ankle, the percentage agreement was >96% and the level of reliability was excellent (ICC_2,1_: 0.93–0.97) across all represented gait speeds. The hip worn AG-DF showed a percentage agreement of 78% at gait speeds between >0.6 and 1.0 m/s but dropped drastically for gait speeds < 0.6 m/s. The reliability was poor (ICC_2,1_: 0.00–0.12) for all gait speeds using the hip worn AG-DF. Conversely, when the hip worn AG-LFEF was applied, the percentage agreement was >88% and the reliability was moderate to good (ICC_2,1_: 0.58–0.88) for gait speeds ranging 0.2–1.0 m/s.

**Figure 3:**
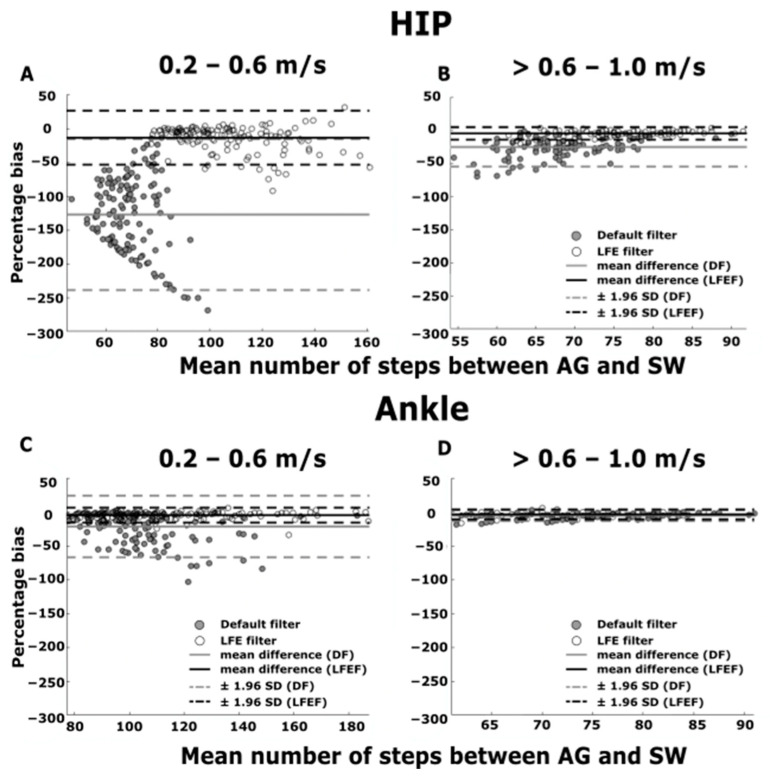
Bland–Altman plots for percentage bias between the hip worn AG and SW vs. the mean number of steps between the hip worn AG and SW using the DF and LFEF for gait speeds between (**A**) 0.2–0.6 m/s. (**B**) >0.6–1.0 m/s during continuous turning. Bland–Altman plots for percentage bias between the ankle worn AG and SW vs. the mean number of steps between the ankle worn AG and SW using the DF and LFEF for gait speeds between (**C**) 0.2–0.6 m/s. (**D**) >0.6–1.0 m/s during continuous turning. The grey and white filled circles represent the DF and LFEF, respectively. The grey and black solid line represents the mean percentage bias for the DF and the LFEF, respectively. The grey and black dashed line represents the ± 95% limits of agreement.

The Bland–Altman plots for walking with continuous turns showed a low mean percentage bias (−3.0–−5.0%; Figure 3C,D) for both ankle worn AG-DF and AG-LFEF for all represented gait speeds (0.2–1.0 m/s) except when using the ankle worn AG-DF at gait speeds between 0.2 and 0.6 m/s (−21.0%; Figure 3C). The mean percentage bias was low for the hip worn AG-LFEF (−4.0%) for gait speeds between >0.6 and 1.0 m/s and otherwise high at all represented gait speeds (−13–−127%; Figure 3A,B).

## 4. Discussion

The purpose of this study was to determine the accuracy of ankle and hip worn AG accelerometers to detect steps using the DF and LFEF as a function of gait speed during steady state walking and continuous turning. The results showed the ankle worn AG-DF to be the most sensitive for step detection at gait speeds > 0.6 m/s, whilst accurate step detection for gait speeds < 0.6 m/s were only observed when applying the LFEF. The hip worn AG-DF showed poor accuracy (12–78%) at gait speeds < 1.0 m/s whereas the accuracy increased to >87% for gait speeds < 1.0 m/s when applying the LFEF. Walking straight in a steady state or while walking with continuous turns did not impact the sensitivity of AG to detect step counts.

While previous studies [8,9,20] have shown the accuracy of AG to determine steps to be poor during slow walking, no previous study have explored at what level the accuracy drops when walking straight and performing continuous turns. In line with previous findings by Treacy et al. (2017), our results showed the hip worn AG-DF to have acceptable percentage agreement with SW for gait speeds > 1.0 m/s but significantly under counts the number of steps at gait speeds between 0.2 and 1.0 m/s for both straight walking and continuous turning. Irrespective of walking condition, the ankle worn AG-DF showed poor accuracy for step detection at gait speeds < 0.6 m/s and an increased accuracy and level of reliability for gait speeds between >0.6 and 1.4 m/s. These findings are in agreement with Treacy et al. (2017), Klaasen et al. (2016), Weber and St. John (2016) and Hergenroeder et al. (2018) who found that ankle worn accelerometers (i.e., SW, Fitbit and AG ankle) are generally more accurate when compared to sensors placed at the hip. This is likely due to the criteria of the AG step detection algorithm, which depends on a signal amplitude threshold of the vertical acceleration to determine if a step is taken. The ground impact and the signal amplitude picked up by the AG generally decreases from the distal to proximal placement (i.e., higher at ankle compared to the hip) [20], which is a plausible explanation for why ankle worn accelerometers overall show greater accuracy for step detection than hip worn sensors. In contrast, the acceleration signal at the hip during slow speeds is most likely not sufficient to register a step using the existing algorithms developed for AG [9].

Walking is an important marker for health where the number of steps per day is often associated with cardiovascular health [1]. Moreover, slow walking speeds has been linked with various movement disorders and high risk of morbidity and mortality [11]. Therefore, it is important to be able to accurately detect steps at low gait speeds to be able to identify health risk. This is especially important for people with disability, who often have compromised gait speeds. Previous studies [8,30] have shown improved accuracy for detecting steps using AG when applying the LFEF. For example, Weber and St. John [8] compared the accuracy of hip and ankle worn AG to an ankle reference sensor in older adults during straight walking [8]. Their results showed the absolute percentage error decreased from 47% to <3% for the ankle worn AG and from 96% to 19% for the hip worn AG when applying the LFEF. In our study, the overall step detection sensitivity for hip and ankle worn AG improved while applying the LFEF, especially at gait speeds < 0.6 m/s. Currently, there is no consensus regarding which gait speeds and (or) frequency movement the LFEF should be applied at. Our results suggest the LFEF should be used at gait speeds < 0.60 m/s for the ankle worn AG and <1.0 m/s for the hip worn AG. On the other hand, walking in daily life often occur in different speed ranges with most people varying their gait speed depending on the purpose of taking steps. Therefore, using the DF and LFEF is a bit more challenging for populations that walk both slow and at normal speeds. In line with this, it is worth noting, that previous studies [18,19,24,31], which recorded accelerometer data over a few days in daily living, have shown the LFEF to overestimate the number of steps taken over a day. Since the LFEF increases the sensitivity at low frequency movements, the AG might be prone to falsely detect steps during stationary movements not related to walking and especially the hip worn AG. Therefore, exploring and redefining the accelerometer amplitude cut points at low frequency movements to negate potential overestimation in daily living is warranted. We suggest that future work should also entail validating the present findings in daily living and to determine a cut-off gait velocity at which the LFEF should be used.

Study limitations include the relatively small sample size including healthy adults that walked slower than their self-selective gait speeds. It is unclear whether healthy controls walking at slower gait speeds reflect the walking pattern of individuals who walk slowly due to old age or disability (e.g., stroke or Parkinson’s). Therefore, future work entails validating the findings in these populations. The study measured steps over a relative short distance and measuring a longer walking distance could result in an increased reliability. On the other hand, the variability of the gait pattern among healthy adults is low and approximately 30 steps has shown to be sufficient for reliable measures of spatial and temporal gait parameters [32]. Therefore, we do not believe our result would have been different if we had assessed a longer duration of walking. Finally, our study included relatively small number of data points at gait speeds > 1.0 m/s. This could result in misrepresentation of the percentage agreement and interclass correlation coefficient at gait speeds > 1.0 m/s. Still, previous studies has shown good reliability of accelerometers at gait speeds > 1.0 m/s [5].

## 5. Conclusions

Our results showed the hip worn AG device to have poor agreement and reliability at gait speeds of <1.0 m/s, with the LFEF drastically increasing the accuracy of the step count at gait speeds between 0.2 and 1.0 m/s. The ankle worn AG showed the highest accuracy at gait speeds > 0.60 m/s, however at gait speeds < 0.60 m/s the LFEF needs to be applied to heavily negate underestimating. Walking straight in a steady state or while walking with continuous turns did not impact the sensitivity of AG to detect step counts.

## Figures and Tables

**Table 1:** Mean percentage agreement (standard deviation) between SW activity monitor and ankle and hip worn AG using the DF and LFEF for different gait speeds during straight walking and walking with continuous turns.

	Straight Walking				Continuous Turning			
Gait Speed (m/s)	Ankle		Hip		Ankle		Hip	
	DF	LFEF	DF	LFEF	DF	LFEF	DF	LFEF
0.2–0.6	71 (19)	96 (7)	12 (17)	87 (22)	82 (17)	96 (5)	25 (23)	88 (15)
>0.6–1.0	96 (5)	97 (5)	69 (25)	97 (5)	95 (4)	97 (4)	78 (13)	96 (5)
>1.0–1.4	96 (4)	97 (4)	92 (7)	97 (4)	-	-	-	-
>1.4	92 (8)	94 (8)	94 (12)	96 (11)	-	-	-	-

**Table 2:** ICC_2,1_ between SW activity monitor and the ankle and hip worn AG using the DF and the LFEF for different gait speeds during straight walking and walking with continuous turning.

	Straight Ahead Walking				Continuous Turning			
Gait Speed (m/s)	Ankle		Hip		Ankle		Hip	
	DF	LFEF	DF	LFEF	DF	LFEF	DF	LFEF
0.2–0.6	0.29	0.97	0.00	0.50	0.28	0.97	0.00	0.58
>0.6–1.0	0.79	0.83	0.00	0.86	0.89	0.93	0.12	0.88
>1.0–1.4	0.85	0.86	0.58	0.87	-	-	-	-
>1.4	0.70	0.70	0.42	0.57	-	-	-	-

## Data Availability

The data used in this study are available from the authors upon request.
